# Factors associated with hyperhomocysteinemia in relatively healthy adult males in northern Shaanxi, China: A cross-sectional study

**DOI:** 10.1097/MD.0000000000042377

**Published:** 2025-05-02

**Authors:** Huihui Guo, Rui Song

**Affiliations:** a Health Management Center, Yulin Hospital, the First Affiliated Hospital of Xi’an Jiaotong University, Yulin City, Shaanxi, China; b Department of Endocrinology, Yulin Hospital, the First Affiliated Hospital of Xi’an Jiaotong University, Yulin City, Shaanxi, China.

**Keywords:** alpha fetoprotein, cardiovascular diseases, height, hyperhomocysteinemia, methylenetetrahydrofolate reductase, thyroid stimulating hormone, uric acid

## Abstract

The elevated level of homocysteine (Hcy) is considered to be a risk factor for cardiovascular diseases and many other diseases, and cardiovascular diseases occurs more frequently in men. The purpose of our study was to evaluate the factors associated with hyperhomocysteinemia (Hhcy) in relatively healthy adult males in northern Shaanxi. This is a cross-sectional study involving male participants who underwent health examinations at the Health Management Center of Yulin Hospital, the first affiliated Hospital of Xi’an Jiaotong University in 2023. Hhcy was defined as plasma homocysteine ≥15 mmol/L. The related factors of Hhcy were analyzed by univariate and multivariate logistic regression. A total of 728 adult males were enrolled, with a median age of 36 years, of whom 274 (37.6%) had Hhcy. Multivariate logistic regression analysis showed that height, methylenetetrahydrofolate reductase TT genotype (hereinafter referred to as TT genotype), uric acid, thyroid stimulating hormone and alpha fetoprotein were significantly associated with Hhcy. In this cross-sectional study, height, TT genotype, uric acid, thyroid stimulating hormone and alpha fetoprotein were significantly associated with Hhcy in relatively healthy adult males in northern Shaanxi.

## 1. Introduction

Homocysteine (Hcy) is an intermediate of protein metabolism, and its only known source is the essential amino acid methionine.^[1]^ Previous studies have shown that heredity, age, sex, nutrition, lifestyle, disease and other factors can lead to the accumulation of Hcy in the body, resulting in the increase of blood Hcy level, which is called hyperhomocysteinemia (Hhcy). According to the Global Burden of Diseases, the global prevalence of cardiovascular diseases (CVDs) has almost doubled in the past 30 years, from 271 million in 1990 to 523 million in 2019. The number of deaths from CVDs increased steadily from 12.1 million in 1990 to 18.6 million in 2019.^[2]^ Hhcy increases the risk of CVDs. The Framingham study found that the risk of stroke in people with Hcy ≥14.24 μmol/L was 1.8 times higher than that in people with Hcy <9.25 μmol/L.^[3]^ Cioni G et al found that Hcy ≥15 μmol/L was significantly associated with coronary artery disease severity (odds ratio [OR], 5.2; 95% confidence interval [CI], 4.8–34.1; *P* = .005) and the presence of atherosclerotic extracoronary lesions (OR, 13.9; 95% CI, 6.2–31.2; *P* < .001).^[4]^

Preventive measures are often more effective than treatment after disease onset. In view of the fact that most of the previous studies on Hcy are aimed at the correlation between Hcy and disease, but there are few studies on the influencing factors of Hcy in healthy people, the purpose of this study is to evaluate the factors related to Hhcy in relatively healthy adult men in northern Shaanxi.

## 2. Materials and methods

### 2.1. Subjects

This is a cross-sectional study involving male participants who underwent health examinations at the Health Management Center of Yulin Hospital, the first affiliated Hospital of Xi’an Jiaotong University in 2023. The basic information collected included age, height, weight, systolic blood pressure and diastolic blood pressure. Fasting blood collection test included Hcy, methylenetetrahydrofolate reductase (MTHFR) genotype, alanine aminotransferase, fasting plasma glucose, glycosylated hemoglobin (HbA1c), uric acid (UA), serum creatinine (Scr), total cholesterol, triglyceride, low density lipoprotein cholesterol, high density lipoprotein cholesterol, thyroid stimulating hormone (TSH), free triiodothyronine, free thyroxine, and alpha fetoprotein (AFP).

The information of 899 health examiner was collected, and 114 cases of abnormal thyroid function were excluded (including 7 cases of hyperthyroidism, 3 cases of subclinical hyperthyroidism, 1 case of hypothyroidism, 81 cases of subclinical hypothyroidism, 22 cases of simple free triiodothyronine or free thyroxine abnormality), 43 cases of Scr beyond the normal reference range, 8 cases of significantly elevated blood glucose (HbA1c ≥9%), and 6 cases of obvious abnormal liver function (alanine aminotransferase ≥3 times the normal reference upper limit). Finally, 728 cases were included. The study was approved by the Ethics Review Committee of Yulin Hospital, the first affiliated Hospital of Xi’an Jiaotong University.

### 2.2. Definition of Hhcy, MTHFR, CVDs, GFR, and BMI

Hhcy was defined as a plasma level of Hcy ≥15 μmol/L^[5]^; MTHFR is a key enzyme in folate metabolism during the 1-carbon unit metabolism process. MTHFR gene mutations cause the MTHFR to lose function or reduce activity, leading to impaired methylation and elevated blood Hcy. Monoallelic heterozygous mutations (CT genotype) may cause mild to moderate elevations in plasma Hcy, but homozygous mutations (TT genotype) often cause severe Hhcy. CVDs, also known as circulatory system diseases, are a group of diseases involving the heart and blood vessels, including hypertension, stroke, coronary heart disease, peripheral arterial disease and heart failure, etc. The calculation formula of glomerular filtration rate (GFR) is the Cockcroft–Gault Ccr calculation formula put forward by Dr Cockcroft and Gaul in 1976.^[6]^ Body mass index (BMI) is calculated by dividing weight (kg) by height (m) squared.

### 2.3. Statistical analysis

The data were analyzed by SPSS 25.0 (IBM, Armonk). The measurement data in accordance with the normal distribution were expressed by the mean ± standard deviation, and the *t*-test was used for the comparison between groups, while the median (first quartile, third quartile) was used for those that did not accord with the normal distribution, and the Mann–Whitney *U* test was used for the comparison between groups. The counting data were expressed by the number of cases (constituent ratio), and the chi-square test was used for comparison between groups. Multivariate logistic regression was used in multivariate analysis. *P* < .05 was considered to be statistically significant.

## 3. Results

The baseline characteristics of the study participants are presented in Table 1. The median age of 728 male participants was 36, and the median Hcy was 13.1 μmol/L. Among the 728 participants, there were 274 (37.6%) in the Hhcy group and 454 (62.4%) in the normal Hcy group. The median Hcy values of the 2 groups were 19.8 and 11.6 μmol/L, respectively.

**Table 1:** Baseline characteristics of the study population (n = 728).

Variable	Total (n = 728)	Hhcy (n = 274)	Normal Hcy (n = 454)	*P*
Age (yr)	36 (33, 39)	35 (32, 39)	36 (33, 39)	.049
Height (cm)	173 (170, 177)	173.5 (171, 177.5)	173 (169, 177)	.019
Weight (kg)	77.0 (69.0, 83.4)	77.0 (69.7, 82.8)	77.0 (68.5, 84.2)	.964
BMI (kg/m^2^)	25.3 (23.2, 27.6)	25.1 (23.0, 27.3)	25.4 (23.4, 27.6)	.239
SBP (mm Hg)	127 (117, 136)	126 (117, 136)	127 (118, 136)	.227
DBP (mm Hg)	80 (73, 88)	79 (72, 88)	81 (73, 88)	.514
Hcy (μmol/L)	13.1 (11.0, 17.5)	19.8 (16.3, 29.7)	11.6 (10.3, 12.9)	<.001
MTHFR genotype				
CC (n, %)	203 (27.9%)	45 (16.4%)	158 (34.8%)	<.001
CT (n, %)	352 (48.4%)	87 (31.8%)	265 (58.4%)	
TT (n, %)	173 (23.8%)	142 (51.8%)	31 (6.9%)	
ALT (U/L)	28 (20, 42)	28 (19, 42)	29 (20, 41)	.314
TG (mmol/L)	1.6 (1.1, 2.4)	1.6 (1.0, 2.5)	1.6 (1.2, 2.4)	.431
TC (mmol/L)	4.6 (4.1, 5.3)	4.6 (4.0, 5.3)	4.6 (4.1, 5.3)	.899
HDL-C (mmol/L)	1.2 (1.0, 1.4)	1.2 (1.0, 1.6)	1.4 (1.2, 1.6)	.902
LDL-C (mmol/L)	3.1 (2.6, 3.6)	3.1 (2.6, 3.5)	3.1 (2.6, 3.6)	.717
FPG (mmol/L)	5.4 (5.2, 5.8)	5.4 (5.1, 5.8)	5.5 (5.2, 5.8)	.049
HbA1c (%)	5.60 (5.40, 5.80)	5.55 (5.40, 5.80)	5.60 (5.40, 5.80)	.002
Scr (μmol/L)	79 (73, 85)	82 (76, 86)	78 (72, 84)	<.001
GFR (mL/min/1.73 m^2^)	122 (107, 139)	120 (106, 134)	123 (108, 141)	.023
UA (μmol/L)	380 (328, 429)	393 (348, 444)	371 (319, 423)	<.001
FT3 (pg/mL)	3.27 ± 0.28	3.26 ± 0.27	3.27 ± 0.28	.467
FT4 (ng/dL)	1.35 (1.24, 1.45)	1.35 (1.26, 1.46)	1.34 (1.24, 1.45)	.274
TSH (μIU/mL)	2.20 (1.63, 3.01)	2.33 (1.72, 3.20)	2.15 (1.60, 2.97)	.059
AFP (ng/mL)	2.68 (2.08, 3.53)	2.83 (2.17, 3.78)	2.60 (2.02, 3.37)	.016

AFP = alpha fetoprotein, ALT = alanine aminotransferase, BMI = body mass index, DBP = diastolic blood pressure, FPG = fasting plasma glucose, FT3 = free triiodothyronine, FT4 = free thyroxine, GFR = glomerular filtration rate, HbA1c = glycosylated hemoglobin, Hcy = homocysteine, HDL-C = high density lipoprotein cholesterol, LDL-C = low density lipoprotein cholesterol, MTHFR = methylenetetrahydrofolate reductase, SBP = systolic blood pressure, Scr = serum creatinine, TC = total cholesterol, TG = triglyceride, TSH = thyroid stimulating hormone, UA = uric acid.

Compared with normal Hcy group, the height (*P* = .019), proportion of TT genotype (*P* = .002), Scr (*P* < .001), UA (*P* < .001), TSH (*P* = .059), AFP (*P* = .016) in Hhcy group were higher, while age (*P* = .049), fasting plasma glucose (*P* = .049), HbA1c (*P* = .002) and GFR (*P* = .023) were significantly lower.

Results from the univariate and multiple logistic regression analyses are shown in Table 2. Univariate logistic regression analyses revealed that the height (*P* = .039), TT genotype (*P* < .001), HbA1c (*P* = .005), Scr (*P* < .001), GFR (*P* = .027), UA (*P* < .001), TSH (*P* = .036), AFP (*P* = .023) were significantly associated with Hhcy. In addition, multiple logistic regression analysis showed that height (OR = 1.04, *P* = .042), TT genotype (OR = 21.60, *P* < .001), UA (OR = 1.004, *P* < .001), TSH (OR = 1.23, *P* = .029), AFP (OR = 1.16, *P* = .035) remained independently and significantly associated with Hhcy.

**Table 2:** Univariate and multiple logistic regression analyses of Hhcy.

Variable	Univariate logistic regression		Multiple logistic regression	
	OR (95% CI)	*P*	OR (95% CI)	*P*
Age (yr)	0.99 (0.97, 1.01)	.220 22	0.98 (0.94, 1.01)	.126 22
Height (cm)	1.03 (1.00, 1.06)	.039	1.04 (1.00, 1.08)	.042
MTHFR genotype				
CC (n, %)	1.00		1.00	
CT (n, %)	1.15 (0.77, 1.74)	.497	1.28 (0.83, 1.97)	.258
TT (n, %)	16.08 (9.65, 26.80)	<.001	21.60 (12.35, 37.78)	<.001
FPG (mmol/L)	0.80 (0.63, 1.02)	.070	0.76 (0.51, 1.13)	.171
HbA1c (%)	0.58 (0.39, 0.85)	.005	0.83 (0.44, 1.56)	.563
Scr (μmol/L)	1.04 (1.02, 1.06)	<.001	1.02 (0.99, 1.06)	.126
GFR (mL/min/1.73m^2^)	0.99 (0.99, 1.00)	.027	0.99 (0.98, 1.00)	.133
UA (μmol/L)	1.004 (1.002, 1.006)	<.001	1.004 (1.002, 1.007)	<.001
TSH (μIU/mL)	1.17 (1.01, 1.36)	.036	1.23 (1.02, 1.48)	.029
AFP (ng/mL)	1.14 (1.02, 1.28)	.023	1.16 (1.01, 1.33)	.035

AFP = alpha fetoprotein, CI = confidence interval, FPG = fasting plasma glucose, GFR = glomerular filtration rate, HbA1c = glycosylated hemoglobin, MTHFR = methylenetetrahydrofolate reductase, OR = odds ratio, Scr = serum creatinine, TSH = thyroid stimulating hormone, UA = uric acid.

## 4. Discussion

In this cross-sectional study, based on health examination records on relatively healthy adult males revealed a number of factors were associated with Hhcy, including height, TT genotype, UA, TSH, and AFP.

This study found that height was an independent risk factor for Hhcy in relatively healthy men, and the risk of Hhcy increased by 4% for every 1 cm increase in height. There have been few similar reports in the past, but a study of 12 to 15-year-olds in Taiwan found that boys’ height, weight and BMI were positively correlated with Hcy.^[7]^ The mechanism has not yet been elucidated. The author speculates that folic acid, vitamin B6 and vitamin B12 are not only related to the metabolism of Hcy, but also to human growth and development. Once the relationship between height and Hcy is confirmed, we expect to see different normal Hcy reference ranges for people in different height groups.

MTHFR is a key enzyme in folic acid metabolism, and MTHFR gene mutation is the most common genetic cause of Hhcy. After adjusting for other risk factors, we found that TT genotype carriers had a 20-fold increased risk of Hhcy compared with CC genotype carriers (OR = 21.60, *P* < .001). This is consistent with previous studies. As early as 1998, Nathalie et al found that Hcy levels in TT genotype carriers were significantly higher than those in CT or CC genotype carriers,^[8]^ and MTHFR gene mutations (CC to TT) aggravated Hhcy in hemodialysis patients.^[9]^

In this study, there were 173 subjects with TT genotype, accounting for 23.8% of the total study population. This value is close to that of other studies. A survey of more than 10,000 Han Chinese by Yang et al found that the TT genotype accounted for 23.2% of the whole population, and the proportion reached 29.3% in males.^[10]^

In this study, it was found that UA was also an independent risk factor for Hhcy in relatively healthy men, for each increase in μmol/L of UA, the risk of Hhcy increased by 0.4%. Many previous studies have pointed out that there is a significant positive correlation between UA and Hcy.^[11-13]^ At present, its mechanism has not been fully elucidated, but considering that UA and Hcy metabolism are related to adenosine, an intermediate product of adenine nucleotide metabolism, Malinow et al proposed that adenosine originating from S-adenosyl-homocysteine, if preferentially incorporated into a precursor pool for UA, would link the syntheses of Hcy and UA.^[14]^

Our study found that the increase of TSH within the normal reference range was an independent risk factor for Hhcy. For each increase in μIU/mL of TSH, the risk of Hhcy increased by 23%. Previous studies have described the correlation between TSH and Hcy, but most of the subjects are people with abnormal thyroid function. The studies of CATARGI^[15]^ and Orzechowska-Pawilojc et al^[16]^ both show that the value of TSH was positively correlated with Hcy in hypothyroidism patients. This may be due to hypothyroidism that affects folate metabolism and enzymes involved in Hcy remethylation, especially MTHFR.^[17]^ However, study by Zou et al^[18]^ showed that TSH is also an independent risk factor for Hcy in people with normal thyroid function, which is consistent with the results of our study. Ding et al found that impaired thyroid hormone sensitivity is associated with elevated Hcy levels in people with normal thyroid function.^[19]^ The Indices of Thyroid Hormones Sensitivity in this paper include the TSH index and the thyrotropin thyroxine resistance index, etc. We calculated the 2 indices in our study and found that the TSH index and thyrotropin thyroxine resistance index of patients with Hhcy were significantly higher than those with normal Hcy, and the difference was statistically significant (not listed in the table). Therefore, we consider that the increase of TSH in our study is more due to the impairment of thyroid hormone sensitivity than the decrease of thyroid function.

Our study found that for each increase in ng/mL of AFP, the risk of Hhcy increased by 16.1%. In the past, there are few studies to explain the correlation between the 2 AFP and Hcy, and the only connection that can be found is the elevation of both in the amniotic fluid of mothers with fetal neural tube defects.^[20,21]^ It is expected that there will be more basic research to explore the correlation between the 2 in the future.

The advantage of this study is that the subjects come from the same unit in the same area, their living and eating habits are similar, and they are all young and middle-aged men with good homogeneity. The drawback is that this is a cross-sectional study, which fails to determine the causal relationship between Hhcy and these risk factors, and does not collect the subjects’ history of tobacco, alcohol and medication.

## 5. Conclusions

Hhcy was found to be associated with height, TT genotype, UA, TSH, and AFP. Further studies are necessary to clarify the relationship between height, UA, TSH, AFP and Hhcy and their role in predicting CVDs.

## Author contributions

**Conceptualization:** Huihui Guo.

**Data curation:** Huihui Guo.

**Formal analysis:** Huihui Guo.

**Investigation:** Rui Song.

**Methodology:** Huihui Guo.

**Project administration:** Huihui Guo.

**Resources:** Huihui Guo.

**Software:** Huihui Guo.

**Supervision:** Huihui Guo.

**Validation:** Rui Song.

**Visualization:** Rui Song.

**Writing – original draft:** Huihui Guo.

**Writing – review & editing:** Huihui Guo.
